# A case report of posterior reversible encephalopathy syndrome caused by ANCA-associated vasculitis case report and retrospective analysis

**DOI:** 10.1097/MD.0000000000032178

**Published:** 2022-12-09

**Authors:** Lulu Dong, Lulu Wang, Chao Jiang, Shuang Li, Minxia Geng, Jiahao Xing, Yajun Chang, Yingying Tian, Rongfang Feng, Tianjun Wang

**Affiliations:** a Graduate School of Hebei North University, Zhang Jiakou, China; b Department of Neurology, Hebei General Hospital, Shijiazhuang, China; c Graduate School of North China University of Science and Technology, Tangshan, China; d Hebei Medical University, Shijiazhuang, China.

**Keywords:** ANCA-associated vasculitis, disturbance of consciousness, PRES, visual impairment

## Abstract

Posterior reversible encephalopathy syndrome is a reversible syndrome clinically manifested by significant variations in neuroimaging. Early treatment is indispensable to achieve the reversibility, which requires the punctual alleviation of its inducing factors.

**Patient concerns:**

Most symptoms of posterior reversible encephalopathy syndrome (PRES) patients can be dispelled followed by a good prognosis after the inducement removal. The patient died due to the untimely diagnosis and treatment of the primary disease. Therefore, sufficient attention should be paid to the PRES induced by ANCA-associated vasculitis (AAV).

**Diagnosis and interventions:**

The patient is a middle-aged male, with acute onset, previous history of hypertension, cholecystectomy, intestinal obstruction, spontaneous renal rupture, etc. For this complaint, the manifestations are mainly blurred vision with convulsion and transient disturbance of consciousness. In 1 day of treatment, these symptoms were relieved, and he was diagnosed as PRES combined with the cranial imaging. The AAV of the patient was confirmed by spleen pathology.

**Outcomes:**

Despite the relief of most symptoms in 1 day of symptomatic treatment, it is highly likely that the patient eventually died of AAV, it is highly likely that the patient eventually died of AAV which will invade the vascular system due to the failure to treat the primary disease in time.

**Lessons:**

For patients diagnosed as PRES, the punctual identification of cause should be performed, so as to diagnose and correct the cause and primary disease as soon as possible, accompanied with the dynamical observation of the relevant indicators for suspected patients to avoid systemic organ failure.

## 1. Introduction

Posterior reversible encephalopathy syndrome (PRES) is characterized by headaches, seizures, visual abnormalities, impaired consciousness and focal neurological deficits, which could result in permanent brain damage and even death in severe cases. The imaging of the disease is dominated by cerebral white matter lesions, commonly observed in the parietal and occipital lobes, with a small proportion involving the basal ganglia, cerebellum and brainstem. Focal signal suggests cerebral edema of vascular, rare enhancement. Antineutrophil cytoplasmic antibody (ANCA)-associated vasculitis (AAV) as an autoimmune disease has not been previously reported in PRES.

## 2. Case presentation

**Figure 1.**
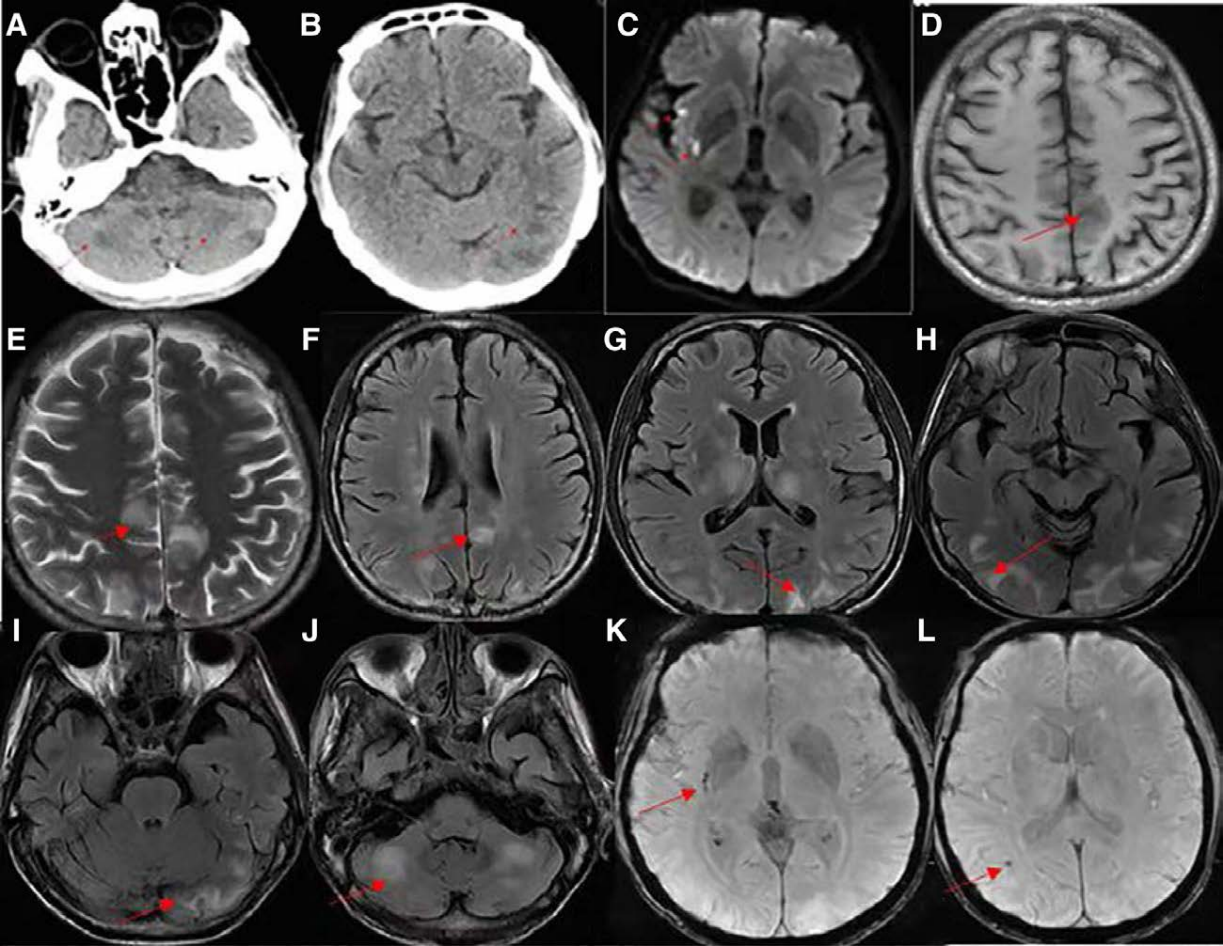
(a, b): CT double frontal-parietal junction cortex, bilateral occipital lobe, bilateral thalamus, and bilateral cerebellar hemisphere lamellar low-density shadow. (c): The right basal ganglia, right insular lobe and abnormal signal shadow of left parietal lobe on DWI?. (d~l): MRI + SWI: d: brain T1W1 symmetrical hypointense shadow in the cerebral hemisphere; (e): symmetrical hyperintense shadow in brain T2W2; (f~j): symmetrical hyperintensities in bilateral basal ganglia, bilateral brain and cerebellar hemisphere of T2Flair brain; (k~l): low signal shadow and micro-hemorrhage in the right basal ganglia area and right temporal occipital junction area of brain SWI. CT = computed tomography, DWI = diffusion-weighted imaging, MRI = magnetic resonance imaging, SWI = sensitive weighted image.

A 45-years-old male was incident of blurred vision in both eyes with unprovoked factors 1 day ago, followed by unconsciousness with Upper-limb spasticity, hanging eyes, stiff trunk, urinary incontinence, which lasted about 1 hour, then consciousness turned clear, with the chief complained of blurred vision. He has undergone the conservative treatment for “intestinal obstruction” in the Department of Gastroenterology, while no meaningful positive results were revealed in examination for rheumatologic and immunologic factors 1 month ago. He reported a history of hypertension of up to 190/110 mm Hg over 20 days, which has not been regularly treated; the “right renal artery embolization” was performed for “spontaneous renal rupture of the right kidney” over 20 days ago. After presented to the emergency department of Hebei General Hospital, the computed tomography (CT) of cerebral indicated a bilateral frontoparietal cortex, bilateral occipital lobes, bilateral thalamus, bilateral cerebellar hemispheres with patchy areas of low density (Fig. 1a, b). Cerebral diffusion-weighted imaging showed the potential of acute or subacute infarction in the right basal ganglia area, right insula the and abnormal signal shadow in the left parietal lobe (Fig. 1c). The inflammatory markers indicated were elevated (leukocyte was 12.93 × 10^9^ cells/L, neutrophil was 11.52 × 10^9^ cells/L, procalcitonin count was 3.714 ng/mL and *C*-reactive protein was 34.86 mg/L). The myocardial was damaged (troponin *T* was 97 ng/L, D-dimer was 9.89 mg/L), as well as the renal (the potassium was 2.8 mmol/L, sodium was 124 mmol/L, urea was 15.11 mmol/L, creatinine was 291 *μ*mol/L, glomerular filtration rate was 21.43 ml/minutes).

The results of examination after admission revealed the blood pressure of 172/115 mm Hg, the decreased visual acuity in both eyes, light perception only, pupils unequal in size bilaterally, left side about 1.0 mm in diameter, right side about 1.5 mm in diameter, muscle strength grade IV in all limbs, and the negative Babinski signs bilaterally. The remaining neurological function was normal according to examination. As a result, the primary diagnosis was cerebral infarction. A combination of nerve nutrition, enlightenment, hypotension, correction of electrolyte disorder and improvement of renal function was administrated. Twenty-four hours after admission the patient’s vision was significantly improved, with the visible finger movements and the possible color recognition. Bilateral pupils were round and equal in size, exhibiting sensitive light reflex, both eyes could freely move in all directions, muscle strength of the limbs was graded as *V*, with positive Babinski’s sign bilaterally. Cerebral magnetic resonance imaging and Sensitive weighted image indicated an acute or subacute lacunar cerebral infarction in the right basal ganglia and symmetrical abnormal signal shadow in the bilateral cerebral and cerebellar hemispheres, as well as microbleeds in the right basal ganglia and the right temporo-occipital junction (Fig. 1d–l). In conjunction with the clinical presentation and imaging, it was diagnosed as PRES.

On the second days of admission, the patient reported the abdominal pain, diarrhea, with vomiting, and pressure and refusal to press on the abdomen in examination. An emergency abdominal CT displayed the splenic rupture and the patient was referred to the hepatobiliary surgery department for “cesarean section & splenectomy.” Postoperatively, he was transferred to the ICU diagnosis of pulmonary infection and type I respiratory failure resulting from wheezing and disturbed acid-base balance. Repeated transfers to ICU were conducted for supportive care considering the unstable condition.

**Figure 2.**
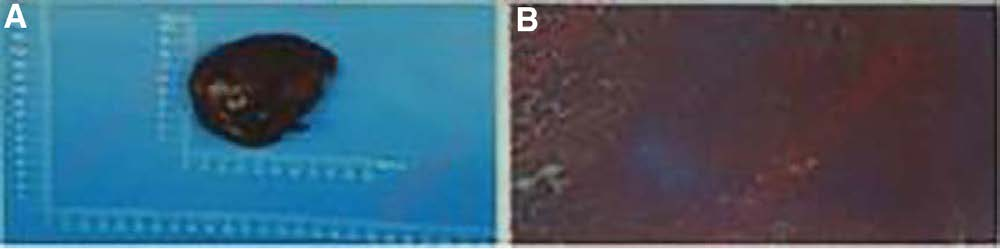
Pathological diagnosis: (Spleen) Hemorrhagic areas were displayed in both gross specimens and microscopically. Multiple fibrinoid necrosis of the vessel wall was seen in the hemorrhagic areas and surrounding splenic tissue, with focal patches of neutrophil, eosinophil and plasma cell infiltration in some vessel walls, perivascular areas, splenic red medullary areas and under the splenic tegument, with microabscess formation and necrosis, and the increased infiltration of surrounding tissue cells. Combined with immunohistochemical staining and serum anti-neutrophil cytoplasmic antibodies (ANCA positive, PANCA positive), this is consistent with ANCA-associated vasculitis. Immunohistochemical staining: -7 slices: Desmin (vessel wall +), SMA (vessel wall +), CD34 (vessel (endothelial +), CD68 (histiocytes +), CD15 (neutrophils +). -2 tablets: Desmin (vessel wall +), SMA (vessel wall +), CD68 (histiocyte +). 10 slices: Desmin (-), SMA (-), CD68 (-). ANCA = antineutrophil cytoplasmic antibody.

Testing report of test results: serum vasculitis test for positive anti-neutrophil cytoplasmic antibodies (perinuclear type), positive myeloperoxidase, splenic pathology suggesting multiple small-vessel fibrinoid necrosis in the spleen with neutrophil infiltration (Fig. 2). Multi-organ failure has successively emerged in the patient, referring to renal failure, heart failure and respiratory failure. According to a hospital-wide multidisciplinary consultation, the patient was considered to suffer from AAV with developed renal rupture developed over 20 days earlier and this splenic rupture, both of which had a certain correlation. Accordingly, he was transferred to the Department of Rheumatology for further treatment. A combination of symptomatic treatment, covering immune boosting with immunoglobulin, nutritional supplementation and anti-infection was administrated, without hormone therapy considering the high index of infection, pulmonary infection and old tuberculosis not excluded by chest CT, and the risk of aggravation of infection and recurrence of tuberculosis. On the second day after transfer, a sudden onset of severe pain in the back of the left shoulder with palpitations and profuse sweating were reported, with the heart rate of 163 bpm, blood pressure of 165/121 mm Hg, anterior with *T*-wave inversion in the ECG, troponin T of 152 ng/L, and amino-terminal brain natriuretic peptide precursor of 6117 pg/mL. Without excluding aortic dissections, the family refused the CT angiography examination of the aorta considering the extremely poor renal function of patient that would be dramatically deteriorated by the application of contrast. Half an hour later, the patient’s heart rate was gradually decreased, without spontaneous breathing, the ECG showed linear and the patient was declared of death. The consideration of the eventual outcome was a high probability of aortic dissections due to AAV invading the coronary system.

## 3. Discussion

Up to now, the specific pathophysiology of PRES has not been completely uncovered, with 3 mechanisms involved a suggested in most studies. In 1 theory, the suddenly rapid development of hypertension exceeds the upper limit of cerebral blood flow autoregulation (180–200 mm Hg) and induces hyperperfusion. When the pressure is rising rapidly and severely, the requirement of autoregulatory response might be beyond the function itself, hyperperfusion can occur, causing the broken blood–brain barrier, allowing the interstitial extravasation of plasma and macromolecules.^[1]^ The hyperperfusion can particularly susceptible in due to the little sympathetic innervation existing there.^[2]^ In conclusion, the persistently elevated blood pressures remain the chief culprit for the clinical symptoms as well as the neurological deficits.

The second mechanism is the endothelial dysfunction induced by a cascade reaction resulting from activation of the immune system. Nearly half of the patients with PRES are equipped with a history of an autoimmune disorder. In most of them, a history of an autoimmune disorder. In most of them, potential chemokines that mediate inflammation are existing, including TNF-*α*, IL-1, IL-6 and INF-*γ*, which can evoke the *T* cell activation and leukocyte adhesion and activation.^[3]^

AAV exists as a group of disorders characterized by inflammation and destruction of small-and medium-sized blood vessels and the presence of circulating ANCA, which can be diagnosed depending on the development of autoantibodies to the neutrophil protein leukocyte proteinase 3 or myeloperoxidase-ANCA. microscopic polyangiitis (MPA) is a type of AAV that can invade small arteries, micro-arteries, capillaries and tiny veins in organs such as the kidneys, skin and lungs, often presenting as necrotizing glomerulonephritis and pulmonary capillaritis. MPA could invade small arteries, microarteries, capillaries and tiny veins in organs such as the kidneys, skin and lungs, generally presenting as necrotizing glomerulonephritis and pulmonary capillarity. myeloperoxidase-ANCA2 in most patients with MPA are positive which can be mainly evoked by the ANCA-mediated over-activation of neutrophils and release of inflammatory cytokines, reactive oxygen species and plasmin.^[4]^ The excessive activation of neutrophils by ANCA could result in the formation of Neutrophil extracellular traps, which are involved not only in ANCA-mediated vascular injury but also in the production of ANCA itself.^[5]^

The third mechanism refers to the anti-diuretic hormone theory proposed by Largeau et al, namely the overproduction of what is known as arginine vasopressin in humans. Lundin et al^[6]^ reported a case of a 62-years-old male with hyponatremia resulting from the prolonged medication for allergic asthma with a serum sodium of 120 mmol/L and positive perinuclear anti-neutrophil cytoplasmic antibodies, ultimately considered to result from the syndrome of inappropriate antidiuretic hormone eliciting eosinophilic granulomatosis and polyangiitis. In addition, Tokushige et al^[7]^ demonstrated that polyangiitis may cause some damage to the hypothalamus or posterior pituitary, inducing arginine vasopressin dysfunction.

In terms of treatment, the removal of etiology lies the main method followed by supportive treatment to delay the progression of the disease.^[8]^ After admission, this patient was provided with symptomatic treatment and nutritional support, which contributed to the recovery of the signs and symptoms of the brain in 1 day. The treatment methods for ANCA-related vasculitis at home and abroad are glucocorticoids, immunosuppressants and plasma exchange, etc.^[9]^ This patient did not receive the above treatment considering the infection, underlying diseases and other factors. In good own condition, whether the symptoms can be improved with better prognosis after the above treatment still requires a lot of clinical research.

The treatment of this case provides a critical basis for exploring the pathogenesis of AAV causing PRES and gives us some insight. Although a negative result on rheumatological and immunological aspects of the gastroenterology screening test were reported 1 month ago, the suspected patient should be performed with dynamical monitor for relevant indicators to avoid progression of the disease to multi-organ failure before treatment that resulted in poor outcome. Early diagnosis of PRES as well as identification of causative factors are therefore essential. There remains various questions to be considered, such as the rate of change of ANCA indicators and the time to the onset of symptoms, and the timing of regular review of suspicious indicators, whether there exist some other mechanisms of action in ANCA-associated vasculitis that affect the systemic immune system requires further study.

## Acknowledgments

The authors would like to thank Tianjun-Wang for her assistance in writing this manuscript.

## Author contributions

All the authors contributed equally to this work. All authors have read and approved the final manuscript.

**Conceptualization:** Lulu Dong, Lulu Wang, Chao Jiang, Shuang Li, Minxia Geng, Rongfang Feng.

**Data curation:** Chao Jiang, Yingying Tian.

**Formal analysis:** Lulu Dong, Shuang Li, Minxia Geng, Yingying Tian.

**Funding acquisition:** Tianjun Wang.

**Investigation:** Lulu Dong, Yajun Chang, Rongfang Feng.

**Methodology:** Lulu Dong, Jiahao Xing.

**Project administration:** Lulu Dong.

**Resources:** Lulu Dong.

**Validation:** Tianjun Wang.

**Visualization:** Lulu Dong.

**Writing – original draft:** Lulu Dong, Lulu Wang, Shuang Li, Jiahao Xing.

**Writing – review & editing:** Lulu Dong, Minxia Geng.
